# Treatment responses for branch retinal vein occlusion predicted by semi-automated fluorescein angiography quantification

**DOI:** 10.1186/s12886-022-02245-w

**Published:** 2022-02-02

**Authors:** Pei-Wei Huang, Chi-Chun Lai, Yih-Shiou Hwang, Wei-Chi Wu, Cheng-Hsiu Wu, Jerry Chien-Chieh Huang, Yen-Po Chen, Laura Liu, Kuan-Jen Chen, Ling Yeung

**Affiliations:** 1 Department of Ophthalmology, Keelung Chang Gung Memorial Hospital, No. 222, Maijin Road, Keelung, 204 Taiwan; 2 Department of Ophthalmology, Linkou Chang Gung Memorial Hospital, Taoyuan, Taiwan; 3 College of Medicine, Chang Gung University, Taoyuan, Taiwan

**Keywords:** Branched retinal vein occlusion, Macular edema, Anti-VEGF, Fluorescence angiography

## Abstract

**Backgrounds:**

Branch retinal vein occlusion (BRVO) is one of the most important causes of visual loss in retinal vascular diseases. The aim of this study is to predict the treatment response of anti-vascular endothelial growth factor (anti-VEGF) therapy in BRVO using semi-automated quantified fluorescein angiography (FA) features.

**Methods:**

This retrospective case-control study enrolled patients with BRVO who are receiving anti-VEGF therapy and have been followed up for > 1 year. Those receiving < 5 anti-VEGF injections in the first year were classified as the responsive group, while those receiving ≥5 injections were the refractory group. The FA images were subjected to semi-automated pre-processing. Fluorescein leakages at the 5-min image were represented by mean gray value over parafoveal and perifoveal regions. FA leakages and central retinal thickness (CRT) on optical coherence tomography (OCT) were used for predicting the treatment response and compared using area under receiver operating characteristic curve (AUC).

**Results:**

Eighty-nine patients (56 males, 33 females, mean age 62.5 ± 10.9 years) with BRVO were enrolled. Of the 89 eyes, 47 (53%) were in the responsive group and 42 (47%) were in the refractory group. The refractory group had a significantly higher number of anti-VEGF injections in the first year (5.9 ± 1.6 versus 2.4 ± 1.2, *p* < 0.001) when compared with that of the responsive group. It had thicker pre-treatment CRT (*p* = 0.011), post-treatment best CRT (*p* < 0.001) and CRT at 1-year (*p* < 0.001). It also had a higher mean gray value over the parafoveal (*p* < 0.001) and the perifoveal (*p* < 0.001) regions. The mean gray value over perifoveal (AUC 0.846) and parafovel (AUC 0.818) had significantly larger AUC than that of the pre-treatment OCT (AUC 0.653; *p* = 0.005 and *p* = 0.016, respectively) when predicting treatment response.

**Conclusion:**

The refractory group had a more severe fluorescein leakage over the parafoveal and the perifoveal regions than the responsive group had. Semi-automated quantified FA leakage can be used as a biomarker for the prediction of anti-VEGF treatment response in macular edema due to BRVO.

## Background

Branch retinal vein occlusion (BRVO) is one of the most important causes of visual loss in retinal vascular diseases [1]. Anti-vascular endothelial growth factor (VEGF) has been used as the standard of care for macular edema secondary to BRVO and could significantly improve the visual outcome [1–3]. However, some patients may suffer from recurrent or persistent macular edema and require repeated anti-VEGF injections [2, 3]. In the BRIGHTER study, the number of anti-VEGF injections varied from a few of injections to almost monthly injections spanning over 2 years [2]. During the 4-year follow-up of the RETAIN study, about 50% of patients had unresolved macular edema [3]. Much effort has been devoted to predicting the treatment response in eyes with BRVO [4–12]. Early switch to intravitreal steroid treatment or applying retinal photocoagulation may be beneficial for some patients [1, 13].

Macular perfusion status could be important in determining the clinical course in eyes with BRVO [9–12]. Fluorescein angiography (FA) and optical coherence tomography angiography (OCTA) were both useful tools for the evaluation of macular perfusion status in different aspect of pathophysiology [14]. Several studies have demonstrated the association between parafoveal vessel density on OCTA and recurrent macular edema in BRVO [6–11]. However, there was limited data on the relationship between FA features and the treatment response [12]. This could be because the quantification of FA images is difficult and potentially biased because of the manual grading involved.

In this study, a semi-automated image processing algorithm to quantify the hyperfluorescence on pre-treatment FA images was designed. The aim was to evaluate whether quantified pre-treatment FA images could help predict the treatment response of anti-VEGF on BRVO-related macular edema.

## Methods

### Patients

Treatment-naïve patients with macular edema secondary to BRVO were included in the current study. All patients were examined at Keelung Chang Gung Memorial Hospital or Linkou Chang Gung Memorial Hospital, and followed up for more than 1 year. All eyes received anti-VEGF treatment, including bevacizumab, ranibizumab or aflibercept, for macular edema under a pro re nata (*PRN*) regimen. After the first anti-VEGF injection, patients were followed up monthly for at least 3 months. Thereafter, the follow-up interval may be gradually extended if the clinical condition was stable. Additional anti-VEGF injections were administered in cases with recurrent or persistent macular edema - defined as central retinal thickness (CRT) ≥ 300 μm. All patients were informed of the off-label use of bevacizumab and provided informed consent prior to treatment. This study was approved by the Institutional Review Board of Chang Gung Memorial Hospital (IRB No.: 202000979B0) and followed the tenets of the Declaration of Helsinki.

The exclusion criteria were as follows: presence of retinal neovascularization within macula; any treatment, including laser photocoagulation, anti-VEGF injection, subtenon or intravitreal steroid injection, received within 3 months prior to FA examination; prior history of pars plana vitrectomy; and the presence of other retinal diseases that could cause macular edema -such as diabetic retinopathy, central retinal vein occlusion (CRVO), hemi-CRVO, age-related macular degeneration, and macular pucker.

### Ocular examination data collection

Demographic data, ophthalmic examination findings, OCT and FA images were collected. Data was collected throughout the clinical course, including the follow-up duration and interval, the number of anti-VEGF injections, the types of BRVO, the types of macular edema, the best-corrected visual acuity (BCVA) at baseline and at 1 year, the central retinal thickness (CRT) on OCT (RTVue-100; Optovue, Inc., Fremont, CA) at pre-treatment, post-treatment, and 1 year. The BRVO was divided into major and macular types based on the definition given in a previous study [15]. Macular edema was classified into 3 different types (cystoid macular edema, diffuse retinal thickening and serous retinal detachment) according to the presentation on OCT. [16] BCVAs were measured using Snellen chart and converted into the logarithm of the minimal angle of resolution (logMAR) for statistical analysis.

### Pre-processing and quantification of FA images

All FA images were obtained using Heidelberg Retina Angiograph 2 (HRA 2; Heidelberg Engineering, Heidelberg, Germany) with a confocal scanning laser ophthalmoscope (cSLO) system for producing images with the resolution of 768 × 768 pixels. Images with 30-degree field of view and with both the macula and the disc included were used in this study.

**Fig. 1:**
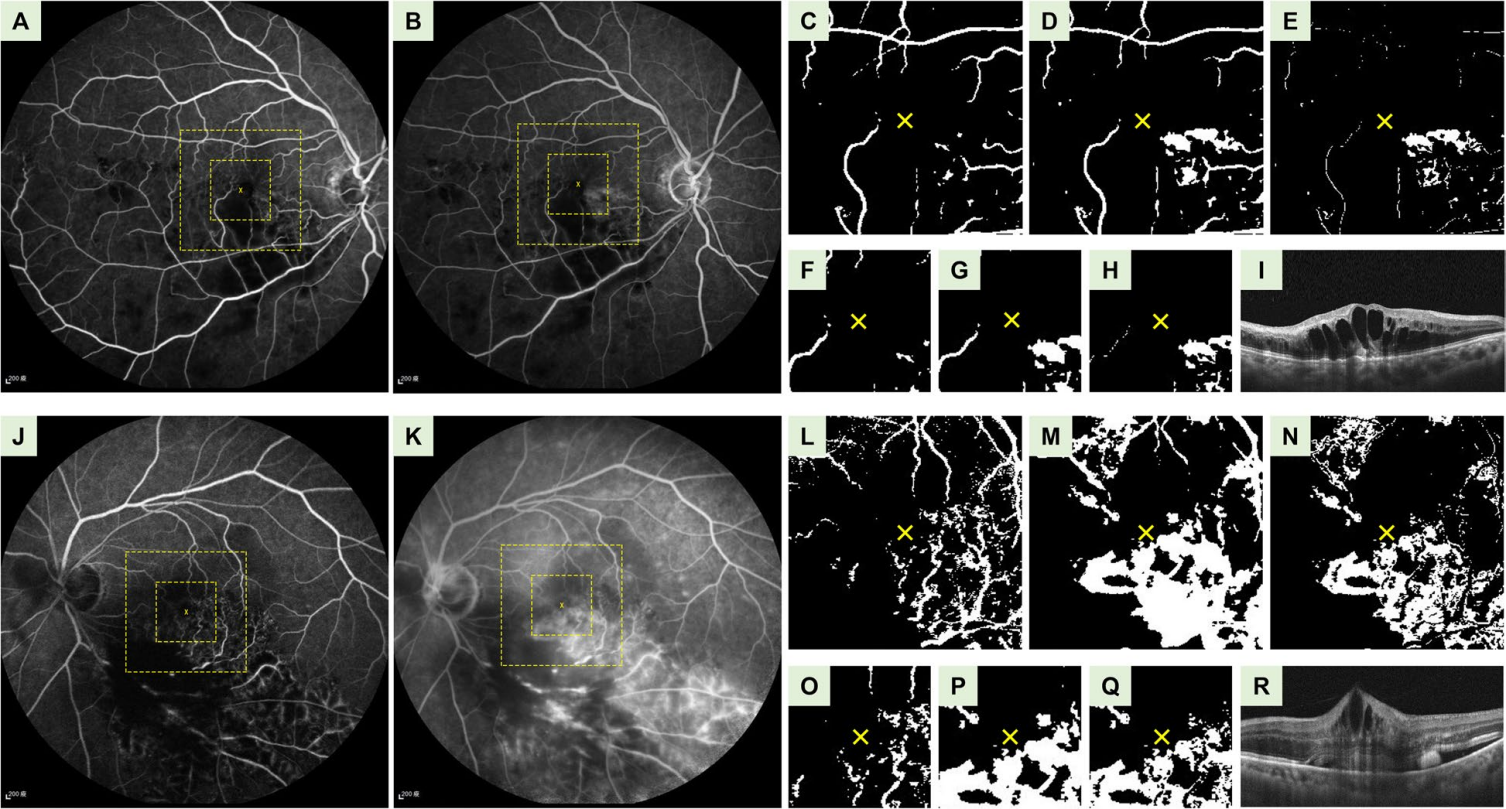
Representative cases in responsive groups (A – I) and refractory groups (J – R). **A**, **J** Fluorescein angiography (FA) images at 1 min. **B**, **K** FA images at 5 min. Large and small squares demarcated by yellow dashed line denote the locations of perifoveal and parafoveal images, respectively. **C**, **L** Binarized FA images of perifoveal area at 1 min. The yellow crosses indicate the location of the foveal centers. **D**, **M** Binarized FA images of the perifoveal area at 5 min. **E**, **N** The perifoveal images after subtracting the image at 1-min from the image at 5-min. Mean gray value over perifoveal region was 13.9 for the responsive case and 63.7 for the refractory case. **F**, **O** Binarized FA images of parafoveal area at 1 min. **G**, **P** Binarized FA images of parafoveal area at 5 min. **H**, **Q** Parafoveal images after subtracting the image at 1-min from the image at 5-min. Mean gray value over the parafoveal image was 8.2 in the responsive case and 48.6 in the refractory case. **I**, **R** Pre-treatment coherence tomography (OCT) images with the central retinal thickness (CRT) of 594 μm in the responsive case and 655 μm in the refractory case

FA images were pre-processed using ImageJ software (Fiji, National Institutes of Health, USA. https://imagej.net/ImageJ) in the following steps (Fig. 1). (1) FA images of each BRVO eye at 1 and 5 min were retrieved. (2) These two images were automatically aligned in a stack using “Linear Stack Alignment with SIFT”. (3) The background intensity gradient was removed using “Subtract Background” (rolling = 50 pixels). (4) Images of 384 × 384 pixels centered at fovea were cropped. (5) Each image was binarized using “Auto Threshold” (method = default). (6) The images were cropped into 240 × 240 pixels and 120 × 120 pixels for calculating the relevant data from the perifoveal and parafoveal regions, respectively. The parafoveal region and perifoveal region were approximately equivalent to the central 3.0 × 3.0 mm region and the 6.0 × 6.0 mm region over the macula. (7) By using the 1-min images as a reference, the fluorescein leakage maps at 5-min were created by subtracting 1-min images from the corresponding 5-min images. (8) The mean gray value, which represents the severity of leakage, was measured in each image. Steps 2–8 could be mostly automatically executed by ImageJ using Macros. A trained grader would only be needed for selecting the location of the foveal center in Step 4.

### Statistical analysis

According to the treatment response, patients were divided into either the responsive or the refractory group based on the criteria modified from a prior study [5]. The responsive group included patients who required < 5 anti-VEGF injections in the first year. The refractory group is comprised of patients who required ≥5 anti-VEGF injections in the first year or had persistent macular edema after 3 monthly anti-VEGF treatment. The differences between the two groups were analyzed using chi-square and independent samples t-test for categorical and continuous data, respectively. Quantitative parameters, including leakages on FA and CRTs, were used for predicting treatment response. The baseline predictive factors that were significantly different (*p* < 0.05) between the responsive group and the refractory group were included in a multivariate analysis using the backward stepwise logistic regression model. The performance of each predictor was then summarized with an area under the receiver operating characteristic (ROC) curve (AUC). The AUCs of quantitative parameters were compared using the Delong test. The pre-processing and quantification of FA images were performed by two independent graders masked for treatment response. The average of the measurements from the two graders was used for statistical analyses in this study. The agreement between the two graders were evaluated by intraclass correlation coefficient (ICC). All of the collected data were analyzed using IBM SPSS Statistics Version 26.0 (IBM Corp., Armonk, NY, USA). Statistical significance was defined using a two-tailed *P* value of < 0.05.

## Results

A total of 89 eyes from 89 patients with BRVO-related macular edema and receiving anti-VEGF treatment were included in this study. Their mean age was 62.5 ± 10.9 years and 63% of the patients were male. Based on the treatment response to anti-VEGF therapy, 47 patients were classified as the responsive group and 42 patients were classified as the refractory group. Table 1 summarizes the demographic data, clinical courses and the quantified FA features in the two groups. There was a strong agreement on the quantified FA features between the two graders. ICC was 0.915 and 0.968 for parafoveal and perifoveal mean gray value, respectively. Compared with those in the responsive group, patients in the refractory group had significantly more anti-VEGF injections in the first year, worse BCVA at 1 year, thicker pre-treatment CRT, thicker CRT at 1 year and thicker post-treatment best CRT. The refractory group also had significantly higher mean gray value over perifoveal and parafovel region. The baseline significant predictive factors such as pre-treatment CRT, mean gray value over parafoveal region and mean gray value over perifoveal region were included in the multivariate regression model. Only mean gray value over perifoveal region (coefficient, 1.144; 95% CI, 1.076–1.216; *p* < 0.001) showed significance in the final model.

**Table 1:** Demographic and clinical data

	**All patients (n = 89)**	**Responsive group (n = 47)**	**Refractory group (n = 42)**	***P* value**
Eye (OD : OS)	49 : 40	26 : 21	23 : 19	0.958
Age	62.5 ± 10.9	60.9 ± 11.7	64.2 ± 9.8	0.160
Sex (male : female)	56 : 33	30 : 17	26 : 16	0.851
Follow-up duration (months)	32.0 ± 21.8	30.0 ± 22.7	34.3 ± 20.8	0.348
Number of anti-VEGF injections in first year	4.1 ± 2.3	2.4 ± 1.2	5.9 ± 1.6	**< 0.001**
Baseline logMAR BCVA	0.88 ± 0.56	0.84 ± 0.60	0.93 ± 0.52	0.416
LogMAR BCVA at 1 year	0.60 ± 0.46	0.50 ± 0.49	0.72 ± 0.40	**0.024**
Pre-treatment CRT (μm)	495 ± 175	451 ± 157	544 ± 183	**0.011**
Post-treatment best CRT (μm)	253 ± 52	233 ± 33	275 ± 61	**< 0.001**
CRT at 1 year (μm)	329 ± 131	252 ± 38	414 ± 146	**< 0.001**
Type of BRVO (major : macular)	61 : 28	30 : 17	31 : 11	0.311
Type of macular edema on OCT				
Cystoid macular edema (yes : no)	47 : 42	21 : 26	26 : 16	0.104
Diffuse retinal thickening (yes : no)	41 : 48	24 : 23	17 : 25	0.317
Serous retinal detachment (yes : no)	13 : 76	6 : 41	7 : 35	0.603
Mean gray value over parafoveal region	25.0 ± 21.1	14.1 ± 12.5	37.3 ± 22.1	**< 0.001**
Mean gray value over perifoveal region	23.3 ± 15.1	14.9 ± 8.9	32.7 ± 15.0	**< 0.001**

Continuous data are presented as mean ± standard deviation

*Abbreviations:* *logMAR BCVA* best-corrected visual acuity expressed in logarithm of the minimal angle of resolution; *BRVO* Branch Retinal Vein Occlusion; *CRT* central retinal thickness; *FA* fluorescein angiography; OCT: optical coherence tomography; *VEGF* vascular endothelial growth factor. Bold values indicate *P* < 0.05

Table 2 includes the AUC of each parameter in predicting treatment response. The mean gray value over perifoveal (AUC 0.846) and parafovel (AUC 0.818) had significantly larger AUC than pre-treatment CRT (AUC 0.653; *p* = 0.005 and *p* = 0.016, respectively) in predicting treatment response.

**Table 2:** Area under receiver operating characteristic curve (AUC) for the prediction of treatment response

	**AUC **	**95% confidence interval**	*P* Value *
Pre-treatment CRT (μm)	0.653	0.540 – 0.767	*reference*
Post-treatment best CRT (μm)	0.739	0.635 – 0.844	0.281
Mean gray value over parafoveal region	0.818	0.732 – 0.905	**0.016**
Mean gray value over perifoveal region	0.846	0.768 – 0.924	**0.005**

*Comparing to pre-treatment CRT using DeLong test

*Abbreviations:* *CRT* central retinal thickness; Bold values indicate *p* < 0.05

**Fig. 2:**
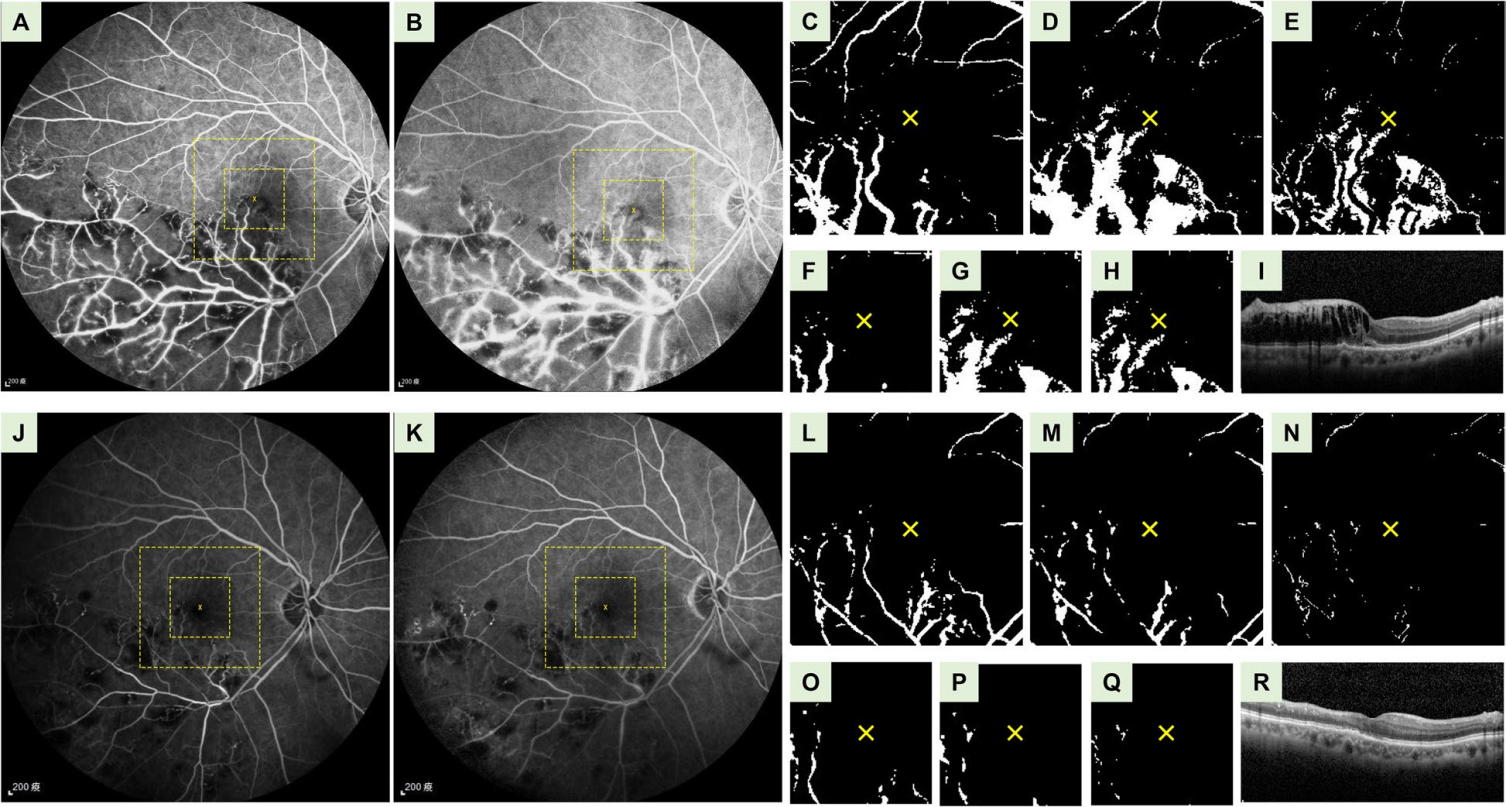
Representative fluorescein angiography (FA) images before (A – I) and after (J – R) anti-VEGF treatment. **A**, **J **FA images at 1 min. **B**, **K** FA images at 5 min. Large and small squares demarcated by yellow dashed line denote the locations of the perifoveal and parafoveal images, respectively. **C**, **L** Binarized FA images of perifoveal area at 1 min. The yellow crosses indicate the location of the foveal centers. **D**, **M** Binarized FA images of the perifoveal area at 5 min. **E**, **N** Perifoveal images after subtracting the image at 1-min from the image at 5-min . The mean gray value over the perifoveal region was 24.3 before treatment and 2.4 after treatment. **F**, **O** Binarized FA images of the parafoveal area at 1 min. **G**, **P** Binarized FA images of the parafoveal area at 5 min. **H**, **Q** Parafoveal images after subtracting the image at 1-min from the image at 5-min. The mean gray value over the parafoveal region was 27.9 before treatment and 1.2 after treatment. **I**, **R** The Central retinal thickness (CRT) on optical coherence tomography (OCT) was 383 μm before treatment and 276 μm after treatment

Fig. 1 demonstrates representative cases in the responsive group **(**Fig. 1**A – I)** and the refractory group **(**Fig. 1**, J – R)**. Patients in the refractory group had more severe leakage on FA. After pre-processing, images from the refractory group had a higher mean gray value over perifoveal and parafoveal region, and a thicker baseline CRT when compared with those from the responsive group. Leakage on FA could be significantly reduced after anti-VEGF treatment; hence, only pre-treatment FA could be used for analysis in the current study. Figure 2 illustrates FA images of the same patient before and after anti-VEGF treatment. When compared with pre-treatment images **(**Fig. 2**A – I)**, post-treatment images **(**Fig. 2**J – R)** had a much lower mean gray value over both the perifoveal and the parafoveal region.

## Discussion

Our study showed that quantified FA leakage over parafoveal and perifoveal regions could be useful for predicting the required number of anti-VEGF injections in eyes with BRVO. Macular perfusion status is important for determining the treatment response in macular edema from BRVO [6–12]. FA and OCTA can evaluate macular perfusion status through different aspects of pathophysiology [14]. OCTA provides high resolution volume-rendering information of retina microvasculature [14]. Capillary non-perfusion on OCTA, either due to complete vascular occlusion or low flow rate (below the detection threshold), could be easily quantified. Reduced macular vessel density, increased macular non-perfusion area, and reduced deep-superficial flow ratio after anti-VEGF treatment were reported to correlate with the recurrence of macular edema [7–11]. However, OCTA artifacts and segmentation errors were commonly found in severe macular edema. Therefore, many of these OCTA parameters were collected after the initial anti-VEGF treatment - when better quality images could be obtained.

FA is important for differential diagnosis, evaluating the macular and peripheral perfusion status, studying the morphology of arteriovenous crossing, and confirming neovascularization in eyes with BRVO [1, 17]. It has good accessibility and has been used frequently in clinical trials and clinical practice [1, 2]. More importantly, FA can evaluate vascular leakage, which reflects the integrity and the functionality of vessels [14]. Apart from the VEGF expression, the pathogenesis of macular edema in BRVO may also involve multiple other pathways such as increased inflammatory cytokine, leukostatsis, alteration of intercellular junction, and impairment of neurovascular unit [18–22]. These mechanisms may increase vascular permeability which could cause fluorescein leakage on FA, but might not be visible on an OCTA. For the same reason, some macular edema may be refractory to anti-VEGF but still respond to intravitreal corticosteroid [13].

Several reports have found that FA classification was associated with visual outcome [23–25] and treatment response in BRVO [12]. However, FA classification is not widely used - probably because it requires manual grading. Manual grading is costly, time-consuming, and involves intra- and inter-rater variability. In this study, all FA images were quantified using semi-automated image pre-processing procedure; the grader was responsible only for retrieving images from the correct time point and locating the foveal center - minimizing potential bias.

**Fig. 3:**
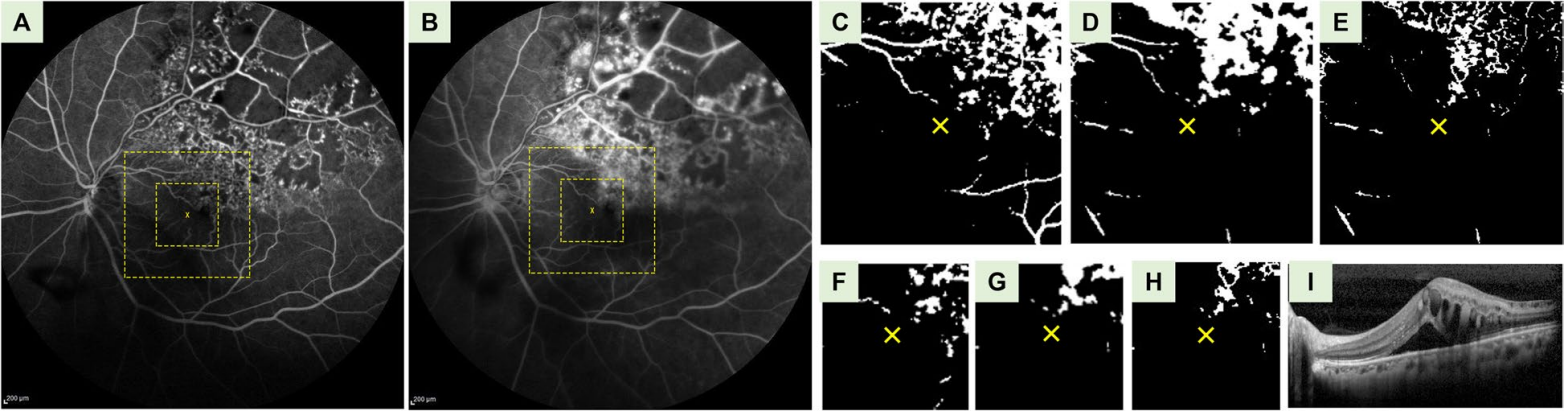
Representative fluorescein angiography (FA) images in responsive group with large non-perfusion area and FA leakage outside the macula. **A** FA images at 1 min. **B** FA images at 5 min. The large and small squares demarcated by yellow dashed line denote the locations of perifoveal and parafoveal images, respectively. **C** Binarized FA images of the perifoveal area at 1 min. The yellow cross indicates the location of the foveal centers. **D** Binarized FA images of the perifoveal area at 5 min. **E** The perifoveal images after subtracting the image at 1-min from the image at 5-min. The mean gray value over the perifoveal region was 13.6. **F** Binarized FA images of the parafoveal area at 1 min. **G** Binarized FA images of the parafoveal area at 5 min. **H** Parafoveal images after subtracting the image at 1-min from the image at 5-min. The mean gray value over the parafoveal image was 14.5. **I** Pre-treatment coherence tomography (OCT) images with a central retinal thickness (CRT) of 702 μm

**Fig. 4:**
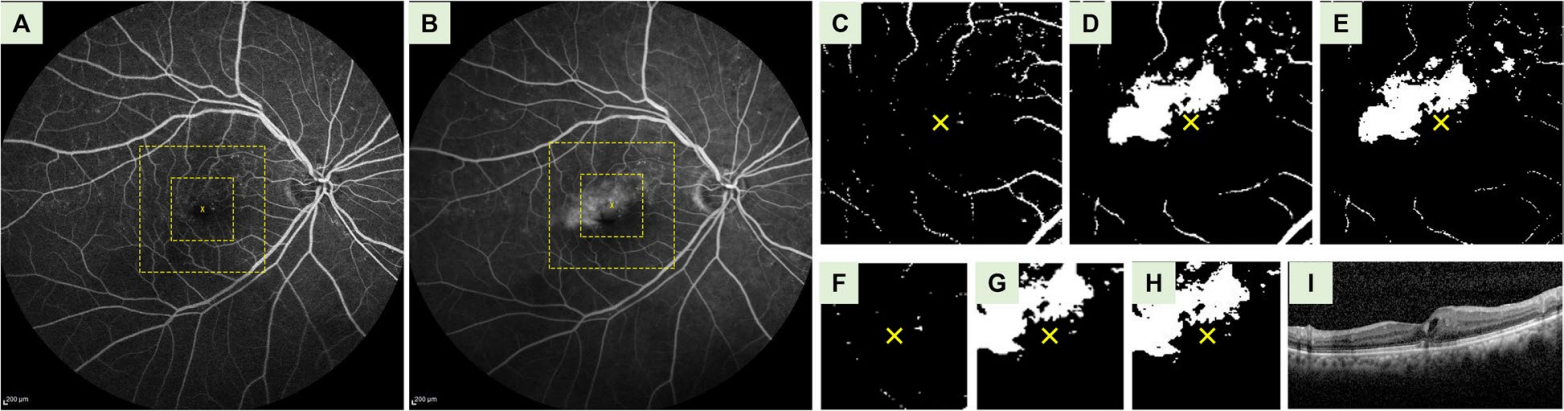
Representative fluorescein angiography (FA) images in refractory group with localized FA leakage over central macula. **A** FA images at 1 min. **B** FA images at 5 min. The large and small squares demarcated by yellow dashed line denote the locations of perifoveal and parafoveal images, respectively. **C** Binarized FA images of the perifoveal area at 1 min. The yellow crosses indicate the location of the foveal centers. **D** Binarized FA images of the perifoveal area at 5 min. **E** Perifoveal images after subtracting the image at 1-min from the image at 5-min. The mean gray value over the perifoveal region was 16.1. **F** Binarized FA images of the parafoveal area at 1 min. **G** Binarized FA images of the parafoveal area at 5 min. **H** Parafoveal images after subtracting the image at 1-min from the image at 5-min. The mean gray value over the parafoveal images was 27.2. **I** Pre-treatment coherence tomography (OCT) images with central retinal thickness (CRT) of 345 μm

Our results suggested that more severe FA leakage corresponds with a higher number of anti-VEGF injections within the first year of treatment. Considering the above pathogenesis of macular edema [18–22], more prominent fluorescein leakage within perifoveal and parafoveal regions may represent more severe injuries to the integrity of the corresponding retinal vessels. The injury could cause refractory or frequently recurrent macular edema. Our results also implied that the location of injured vessel could be an important issue. Figure 3 demonstrates a patient who suffered from BRVO with a large non-perfusion area and severe FA leakage outside macula. However, the mean gray values over perifoveal and parafoveal regions were low (13.6 and 14.5, respectively). The patient responded well to anti-VEGF therapy. After 3 doses of anti-VEGF injections, there was no more recurrence of macular edema. Figure 4 demonstrates another patient who suffered from BRVO with localized FA leakage within the macula. The mean gray value over parafoveal region was low (16.1), but over perifoveal region was high (27.2). After 8 doses of injection within 1 year, there was still recurrence of macular edema. This patient was classified in the refractory group. Our hypothesis is consistent with the clinical observation that while some extramacular BRVO with large non-perfusion area may develop neovascular complications but rarely cause macular edema. Some other BRVO that involved only a small area within the macula may suffer from refractory macular edema.

Our results suggested that the types of BRVO and the types of macular edema were not significantly different between the responsive group and refractory group. When different types of BRVO or different types of macular edema was added as predictive variable in the multivariate logistic regression model, none of them was a significant predictive factor for treatment response.

The 1-min image could show retinal vessels and optic disc morphology, but the dye leakage was minimal at this time point. One-min images were subtracted from 5-min images in image pre-processing. The hyperfluorescent areas in the resulting images consisted mainly of dye leakage during the time lapse. Apart from the 5-min images, we also evaluated the performance of 10- and 15-min FA images by the same image pre-processing method. However, their performance was slightly worse than that of the 5-min images. This could be because 5-min is enough for obvious leakage from vessels with poor integrity, while the ischemic area remains *hypofluorescent*. With further increase in the time lapse (e.g., at 10 or 15 min), the leakage may become too prominent and spread diffusely to both ischemic and non-ischemic areas. Therefore, the 10- and 15-min FA images are less important for the determination of the macular perfusion status, and for the prediction of the treatment response.

Apart from macular perfusion status, CRT on OCT could also be an useful tool for predicting the treatment response of BRVO [4, 5, 12]. Yoo et al. discovered that the baseline CRT could be used to predict the recurrence of macular edema with an AUC of 0.745 [12]. Our study also showed that baseline CRT and post-treatment best CRT may be useful for predicting treatment response in BRVO. However, leakage on FA had significant larger AUCs than that of the baseline CRT.

Moon et al. found that patients with thicker post-treatment CRT were more likely to develop refractory macular edema [5]. Vogl et al. established a machine learning model for predicting macular edema recurrence in 1 year by using sequential OCTs [4]. A high AUC (0.83) could be achieved with this model but the model requires OCT data from the initial three observations. Although the Vogl model’s AUC was similar to the AUC of FA leakage in our study, different predictive information was generated. Instead of predicting recurrence, our study focused on the number of injections which is directly related to the patient’s treatment cost and visiting burden. Our model also only requires pre-treatment FA to run. Our model could help optimize treatment strategy at a very early stage.

The limitations of our study include small sample size and short follow-up duration. However, it provides an innovative way for making refractory macular edema prediction using just the pre-treatment FA images. Currently, there was no consensus on the treatment regimen and re-treatment criteria for BRVO. Owing to retrospective nature of this study, the treatment protocol was not strictly standardized. However, the *PRN* anti-VEGF regimen in our study is compatible with common real-world situations.

In conclusion, this study introduces an innovative semi-automated image processing algorithm to quantify the hyperfluorescence on FA images. Using the quantified pre-treatment FA parameters, patients who are refractory to anti-VEGF therapy could be promptly identified with AUC > 0.8. Early identification of these poor-response patients may potentially guide clinicians in designing personalized treatment strategy, thus reduce treatment burden.

## Data Availability

The datasets used and/or analyzed during the current study are available from the corresponding author upon reasonable request.
